# Supplementary material for: Porous Carbon–Carbon Composite Materials Obtained by Alkaline Dehydrochlorination of Polyvinyl Chloride
Source: Materials (Basel). 2022 Oct 30;15(21):7636. doi: 10.3390/ma15217636 (PMC9653659; doi:10.3390/ma15217636)
Supplement: Supplementary file 1 [file materials-15-07636-s001.zip › materials-1955096-supplementary.pdf]

*Supplementary Materials*

# Porous Carbon–Carbon Composite Materials Obtained by Alkaline Dehydrochlorination of Polyvinyl Chloride

**Yury G. Kryazhev [1], Irina V. Anikeeva [1], Mikhail V. Trenikhin [1,2], Tatiana I. Gulyaeva [1], Valeriy P. Melnikov [3], Vladimir A. Likholobov [4] and Olga B. Belskaya [1,2,*]**

[1] Center of New Chemical Technologies BIC, Boreskov Institute of Catalysis, Siberian Branch, Russian Academy of Sciences, 644040 Omsk, Russia
[2] Petrochemical Institute, Department Chemistry and Chemical Technology, Omsk State Technical University, Omsk 644050, Russia
[3] N.N. Semenov Federal Research Center for Chemical Physics, Russian Academy of Sciences, 119991 Moscow, Russia
[4] Boreskov Institute of Catalysis, Siberian Branch, Russian Academy of Sciences, 630090 Novosibirsk, Russia
* Correspondence: obelska@ihcp.ru or obelska316@rambler.ru; Tel.: +7-(3812)670474

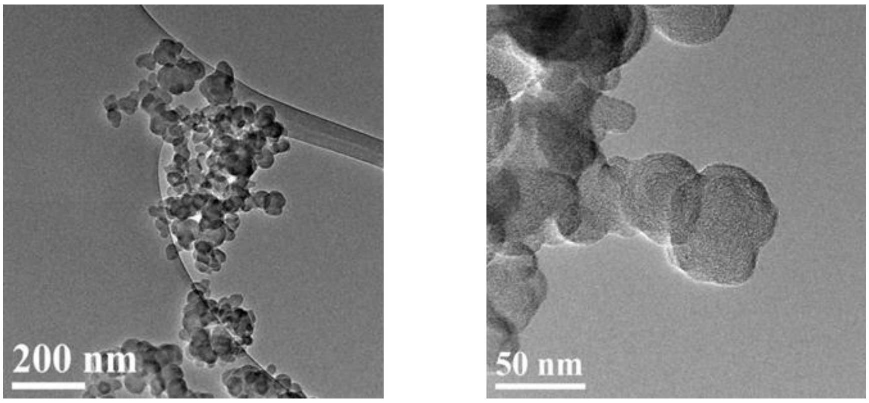


**Figure S1.** TEM image of the aggregates of primary carbon globules typical of P 267-E grade NGC.

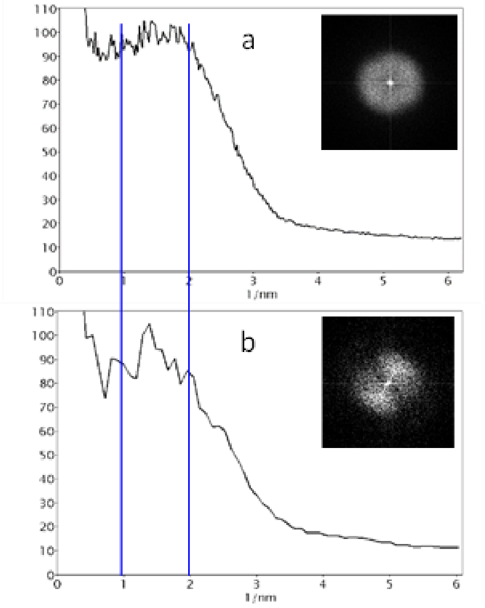


**Figure S2.** A comparison of the radial contrast intensity profiles obtained for the carbon matrix in the samples of CM (a) and PCCNC with incorporated NGC (b) (see Figs. 3d and 4f in the text).
